# Rhamnolipids Nano-Micelles as a Potential Hand Sanitizer

**DOI:** 10.3390/antibiotics10070751

**Published:** 2021-06-22

**Authors:** Marwa Reda Bakkar, Ahmed Hassan Ibrahim Faraag, Elham R. S. Soliman, Manar S. Fouda, Amir Mahfouz Mokhtar Sarguos, Gary R. McLean, Ali M. S. Hebishy, Gehad E. Elkhouly, Nermeen R. Raya, Yasmin Abo-zeid

**Affiliations:** 1 Botany and Microbiology Department, Faculty of Science, Helwan University, Ain Helwan, Cairo 11795, Egypt; marwa_mahmoud01@science.helwan.edu.eg (M.R.B.); professor_ahmed85@science.helwan.edu.eg (A.H.I.F.); 2 Bioinformatics Center, Faculty of Science, Helwan University, Ain Helwan, Cairo 11795, Egypt; 3 Cytogenetics and Molecular Genetics Unit, Botany and Microbiology Department, Faculty of Science, Helwan University, Ain Helwan, Cairo 11795, Egypt; elham_soliman@science.helwan.edu.eg; 4 Biochemistry and Chemistry Department, Faculty of Science, Helwan University, Ain Helwan, Cairo 11795, Egypt; m_fouad80@science.helwan.edu.eg; 5 Biotechnology Department, Faculty of Science, Helwan University, Ain Helwan, Cairo 11795, Egypt; AmirElkoshy@gmail.com; 6 Cellular and Molecular Immunology Research Centre, London Metropolitan University, 166-220 Holloway Road, London N7 8DB, UK; g.mclean@londonmet.ac.uk; 7 National Heart and Lung Institute, Imperial College London, Norfolk Place, London W2 1PG, UK; 8 Chemistry Department, Faculty of Science, Helwan University, Cairo 11795, Egypt; ashebishy@science.helwan.edu.eg; 9 Department of Pharmaceutics, Faculty of Pharmacy, Helwan University, Cairo 11795, Egypt; gehad.elkhouly@pharm.helwan.edu.eg (G.E.E.); Nermeen.Rayh@pharm.helwan.edu.eg (N.R.R.); 10 Helwan Nanotechnology Center, Helwan University, Helwan, Cairo 11795, Egypt

**Keywords:** COVID-19, SARS-CoV-2, rhamnolipids, nano-micelles, *Pseudomonas aeruginosa*, antibacterial agent, antiviral agent, docking studies

## Abstract

COVID-19 is a pandemic disease caused by the SARS-CoV-2, which continues to cause global health and economic problems since emerging in China in late 2019. Until now, there are no standard antiviral treatments. Thus, several strategies were adopted to minimize virus transmission, such as social distancing, face covering protection and hand hygiene. Rhamnolipids are glycolipids produced formally by *Pseudomonas aeruginosa* and as biosurfactants, they were shown to have broad antimicrobial activity. In this study, we investigated the antimicrobial activity of rhamnolipids against selected multidrug resistant bacteria and SARS-CoV-2. Rhamnolipids were produced by growing *Pseudomonas aeruginosa* strain LeS3 in a new medium formulated from chicken carcass soup. The isolated rhamnolipids were characterized for their molecular composition, formulated into nano-micelles, and the antibacterial activity of the nano-micelles was demonstrated in vitro against both Gram-negative and Gram-positive drug resistant bacteria. In silico studies docking rhamnolipids to structural and non-structural proteins of SARS-CoV-2 was also performed. We demonstrated the efficient and specific interaction of rhamnolipids with the active sites of these proteins. Additionally, the computational studies suggested that rhamnolipids have membrane permeability activity. Thus, the obtained results indicate that SARS-CoV-2 could be another target of rhamnolipids and could find utility in the fight against COVID-19, a future perspective to be considered.

## 1. Introduction

COVID-19 (coronavirus disease-2019), a pandemic infection caused by the newly emerged coronavirus, SARS-CoV-2, reported in Wuhan, China [1] and has spread globally with 159,845,155 confirmed cases and 3,321,212 deaths across 192 countries by 12 May 2021 (according to the COVID-19 Dashboard by the Center for Systems Science and Engineering (CSSE) at Johns Hopkins University). This disease is an acute respiratory disorder, characterized by pneumonia, dry cough, fever and body pain with a high rate of mortality, particularly in older people (>80 years) or those with underlying health conditions [2]. Due to the long incubation period of the virus in humans of up to 14 days and airborne transmission via aerosols, the main transmission source of virus is through human to human interactions [3]. Moreover, SARS-CoV-2 is also estimated to remain infectious on solid surfaces for up to 9 days [4], and this has been postulated to facilitate spread of infection by self-inoculation of mucous membranes of the nose, eyes or mouth, similarly to other human coronaviruses [5,6]. 

Due to a lack of standard treatments and identification of mutated variants of SARS-CoV-2 in several countries that might render the developed vaccines ineffective in the future, the WHO has advised a healthy lifestyle and the adoption of proper social distancing measures for infection prevention and control. To help in containing COVID-19 infection, WHO recommends hand washing or sanitation frequently with soap or ≥60–80% alcoholic hand sanitizer, respectively. Alcohol-based hand sanitizers were promoted due to their broad-spectrum antimicrobial activity, their availability and good safety profiles [7].

However, under the current pandemic conditions and the possibility of misuse by people in the community, alcohol-based sanitation was reported to raise several hazards to humans and the environment [8]. For example, dermal contact of ethanol was reported to cause irritation and allergic conditions of skin and eyes with prolonged exposure resulting in dryness or cracking of the skin with peeling redness or itching [7]. Such frequent use of sanitizer, especially during the current pandemic, has been reported to be responsible for skin damage, which reduce its ability to work as a barrier against other harmful pathogens, thus increasing the possibility of further infections by microorganisms [9]. Overuse of alcohol-based hand sanitizers in some cases was also reported to increase the risk of viral outbreaks [9,10], such as, increased risk of norovirus [11].

Furthermore, alcohol-based hand sanitizer use can result in antimicrobial resistance where microorganisms mutate as a defense mechanism to the repeated exposure to genotoxic chemicals, leading to the development of resistant strains and increased burden on already struggling healthcare professionals [9,12]. For example, *Enterococcus faecium* was reported to be 10 times more resistant to alcohol-based hand rubs than older isolates after repeated exposure to alcohol-based sanitizer [13]. It has been also reported that *Escherichia coli* and *Pseudomonas aeruginosa* were 48% and 64% resistant, respectively, against all available sanitizers on the market [14].

Therefore, there is a high demand to find novel alternative approaches for hand sanitation with minimal adverse effects. Biosurfactants are microbially produced surface active compounds [15], characterized by their biodegradability [16], low toxicity [17], low skin irritation potential [18], and have antimicrobial activity against a variety of pathogens [19,20].

**Figure 1:**
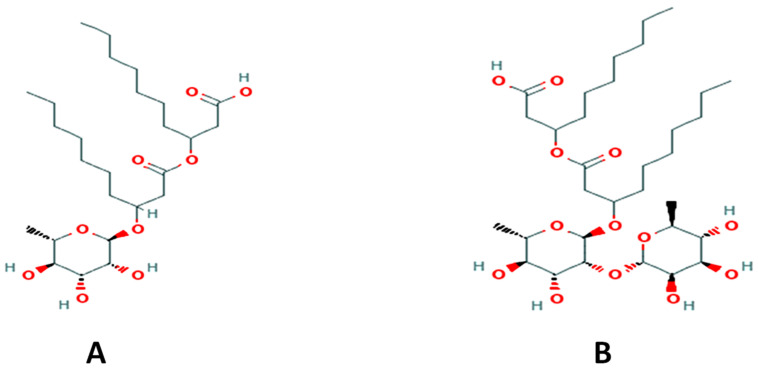
Chemical structure of (**A**) rhamnolipids R1 (mono-rhamnolipids; Rha(s)1) and (**B**) rhamnolipids R2 (di-rhamnolipids; Rha(s)2). Chemical structures were retrieved from PubChem; https://pubchem.ncbi.nlm.nih.gov (accessed on 16 June 2021).

Rhamnolipids (Rha(s)) (Figure 1) are one such biosurfactant reported to have antimicrobial activity with no cytotoxic effect when applied onto rabbit skin [21]. They are economically produced by *Pseudomonas aeruginosa*, a bacterial source with potential to scale-up their production [22].

Rha(s) were previously reported to be effective against multidrug resistant Gram positive and Gram-negative bacteria such as *Escherichia coli, Micrococcus luteus, Alcaligenes faecalis, Serratia marcescens, Mycobacterium phlei* and *Staphylococcus epidermidis*, methicillin-resistant *Staphylococcus aureus* (MRSA) [23], *Staphylococcus aureus* and *Enterococcus faecalis* I27 [24,25]. The antiviral activity of Rha(s) was also reported against herps simplex viruses, HSV1 and HSV2 resulting in virus inactivation in-vitro [26]. The structure of herpes viruses consists of a large double-stranded DNA genome encased within an icosahedral protein capsid wrapped in a lipid bilayer envelope, which also contains embedded viral glycoproteins. It was argued that the antiviral activity of Rha(s) against HSV1 and HSV2 was due to: (1) their ability as a biosurfactant to interact with the lipid membranes of these viruses and (2) the beta hydroxyalkanoic acids found in the structure of Rha(s) inducing changes to the lipid envelop viral glycoproteins [26].

SARS-CoV-2 is also an enveloped virus containing a positive-sense RNA genome [27,28]. The lipid bilayer contains viral spike glycoprotein, whose receptor binding domain (S1- RBD) allows attachment of virus to the host cell receptors, angiotensin converting enzyme-2 (ACE2), essential for virus entry into cells and thus infectivity [29]. Based on the structure similarity with herpes simplex viruses, we assume that Rha(s) can interact with the SARS-CoV-2 lipid envelop and spike glycoproteins rendering them inactive. Based on these findings, we hypothesize that Rha(s) could be a good alternative for alcohol-based hand sanitizers and find utility in the COVID-19 pandemic.

In the current work, Rha(s) were produced by *Pseudomonas aeruginosa* using a new production medium to increase the yield of Rha(s). Rha(s) nano-micelles were then prepared and tested. To ascertain the broad antimicrobial activity of Rha(s) nano-micelles, the antibacterial activity of Rha(s) nano-micelles was first investigated against selected drug resistant bacterial strains: *Staphylococcus aureus*, *Streptococcus pneumoniae*, *Salmonella* Montevideo and *Salmonella* Typhimurium. Subsequently, a docking study was also performed to investigate the potential antiviral activity of Rha(s) against SARS-CoV-2. The docking study was performed to understand the possible interactions of Rha(s) with the S1- RBD and the lipid envelop of SARS-CoV-2. Moreover, the interaction of Rha(s) with the active sites of different enzymes involved in SARS-CoV-2 replication; EndoRNAse, helicase, RNA-dependent RNA polymerase and main protease was also assessed in the docking study. Collectively, these studies aim to determine the possibility of Rha(s) application as a treatment for viral infections. However, their antiviral activity in vitro and safety profile for treatment still requires addressing.

## 2. Results

### 2.1. Isolation and Identification of Bacterial Strain

Initially our bacterial strain LeS3 was isolated and suspected as *Pseudomonas aeruginosa* due to its green fluorescence colonies on agar plates. The PCR amplification products of 16S rRNA encoding gene showed a fragment length of 1500 bp (see Figure S1 in Supplementary Materials). The PCR product sequence was aligned to the bacterial (taxid:2) nucleotide collection of the NCBI database using the (megablast) algorithm. The alignment showed a 99% query coverage with 97% identity to *Pseudomonas aeruginosa*. The 16S rRNA encoding gene sequence of *Pseudomonas aeruginosa* strain LeS3 was deposited to NCBI gene bank with accession number MN960161 (https://www.ncbi.nlm.nih.gov/nuccore/MN960161, accessed on 16 June 2021).

### 2.2. Production and Characterization of Rha(s)

*Pseudomonas aeruginosa* strain LeS3 previously identified in Section 2.1 was cultivated to produce Rha(s) using two different culturing media, namely chicken carcass soup (CCS) and glycerol supplemented nutrient broth (GSNB) in a trial to optimize the yield of Rha(s). The yield of Rha(s) produced in CCS was 5 times (0.5g L^−1^) more than that recovered by GSNB (0.1 g L^−1^). Therefore, all the following experiments and characterization tests were performed on Rha(s) produced using CCS media.

#### 2.2.1. Characterization of Rha(s)

Rha(s) mixture produced from *Pseudomonas aeruginosa* strain LeS3 grown in new designed culture medium (CCS) for 72 h was analyzed using the ESI-MS spectrometer coupled to UPLC (LC/ESI-MS). To confirm the structural composition, mass spectra were acquired in both positive and negative ion modes. Analysis of the obtained mass spectra was performed by calculating the elemental composition depending on acquired knowledge concerning mass spectrometry of the related compounds and comparing the obtained data with those available in the literature [30,31,32,33]. Our data (Table 1) revealed that most spectra peaks were found in the positive pseudo-molecular ion. All Rha(s) congeners appeared in the positive spectra accomplished with adduct ions; [M+H]^+^, [M+K]^+^, [M+Na]^+^ and [M-H+Na_2_]^+^, especially [M+Na]^+^. Only 3 Rha(s) congeners were detected in both positive and negative ion modes. This matched with what was reported previously by Pantazaki and colleagues [33] who attributed these results to the presence of formic acid, methanol and water in the sample.

Rha(s) mixture was found to contain the four major groups of rhamnolipids; mono-rhamnolipid-mono-lipidic, mono-rhamnolipid-di-lipidic (Rha(s)1), di-rhamnolipid-mono-lipidic and di-rhamnolipid-di-lipidic (Rha(s)2). The lipid chains varied between saturated and unsaturated fatty acids. Out of 22 Rha(s) congeners detected by LC/ESI-MS, 17 congeners belonged to rhamnolipids with one rhamnose moiety (Rha(s)1), and 5 congeners belonged to rhamnolipids with two rhamnose moieties (Rha(s)2). The congeners ration of Rha(s)1 to Rha(s)2 was; 94.84%: 3.89%, therefore, Rha(s)1 congeners are the dominating form.

In our study, we obtained a mixture of Rha(s) congeners with a molecular weight ranging from 302 to 815. Among the congeners, Rha(s)1 with the polyunsaturated β-hydroxy-fatty acid chains (R-C_8:2_) was found to predominate with a relative abundance of 29.4% and m/z 325 [M+Na]^+^ followed by, R-C_8:1_, R-C_10_ and R-C_8_, with a relative abundance of 25.2%, 13.6% and 12.8%, respectively. The predominant Rha(s)2 congener was detected at *m/z* 673 [M+Na]^+^ and at *m/z* 649 [M-H]^−^ with a relative abundance of 1.6%. Interestingly, 11 Rha(s) congeners with mono and di unsaturated fatty acid chains were identified in both Rha(s)1 (R-C_8:1_, R-C_8:2_, R,C_9:1_, R-C_10:2_, R-C_12:2_, R-C_13:2_, R-C_8_-C_14:1_, R-C_9_-C_13:1_, R-C_10_-C_12:1_, R-C_11_-C_11:1_, R-C_14_-C_16:2_ and R-C_15_-C_15:2_) and Rha(s)2 (R-R-C_12:1_, R-R-C_16:1_, R-R-C_8_-C_10:2_, R-R-C_9_-C_9:2_ and R-R-C_16_-C_16:2_).

### 2.3. Preparation and Characterization of Rhamnolipids Nano-Micelles

Rha(s) nano-micelles were prepared in phosphate buffered saline, pH 7.4 (PBS, pH 7.4) using a probe sonicator. The particle size and zeta potential were recorded by Malvern zeta-sizer instrument and presented in Table 2 as average diameter (D, nm) ± SD and average Zeta potential (mv) ± SD, respectively. As revealed from Table 2, the size of nano-micelles ranged from 164 ± 1 to 274 ± 50 nm, and all samples had a zeta potential value ≥ −50 mv. The polydispersity index (PDI) value indicated a monodisperse sample (PDI ≤ 0.3) except for nano-micelles prepared at 1 mg mL^−1^ (PDI > 0.3).

**Figure 2:**
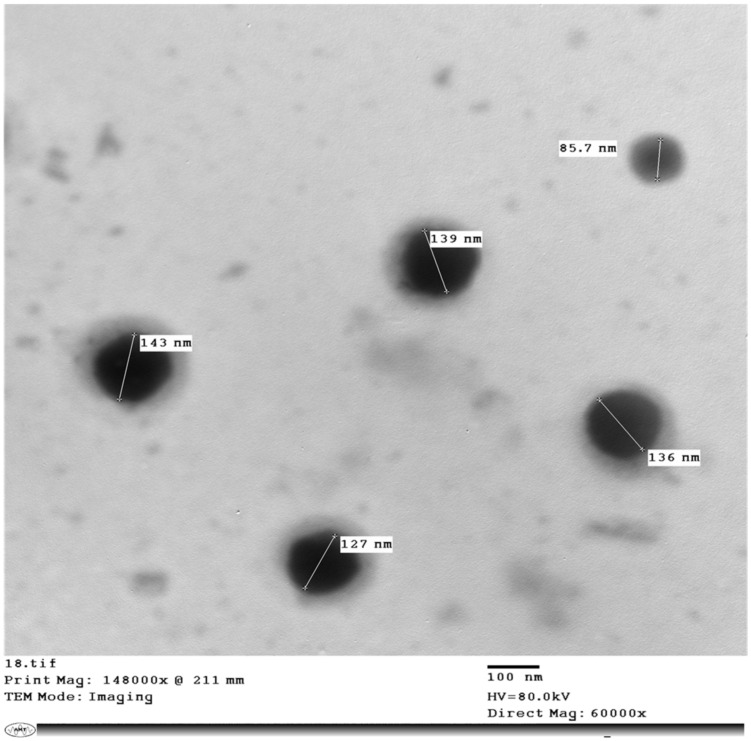
TEM image of rhamnolipids nano-micelles prepared at a concentration of 5 mg mL^−1^.

Transmission electron microscopy (TEM) image of Rha(s) nano-micelles (5 mg mL^−1^) is presented in Figure 2 and shows the particle size ranged from 85.7 to 143 nm. This was reduced slightly more than that recorded by the Malvern zeta-sizer instrument. The TEM image also shows spherical nano-micelles with no sign of aggregation.

### 2.4. Antibacterial Activity of Rhamnolipids Nano-Micelles

The antibacterial activity of Rha(s) nano-micelles against selected human drug resistant bacterial pathogens was performed by agar well diffusion method and results obtained are presented in Table 3. Rha(s) nano-micelles were active against both Gram-positive and Gram-negative bacteria, however, they showed a higher antibacterial activity against Gram-positive bacteria as revealed by minimum inhibitory concentration (MIC) for Gram-positive strains (*Streptococcus pneumoniae* and *Staphylococcus aureus*) that was significantly (*p* < 0.05) lower than MIC for Gram-negative strains *(Salmonella* Montevideo and *Salmonella* Typhimurium). The antibacterial activity of Rha(s) nano-micelles was concentration and size dependent. An increase of nano-micelles concentration was associated with an improvement of the antibacterial activity against both Gram-positive and Gram-negative bacteria as demonstrated by the significant increase (*p* < 0.05) of zone of inhibition diameter in each bacterial strain with increasing concentration (Table 3). Nano-micelles prepared at 5 and 10 mg mL^−1^ had a smaller size, 164 and 169 nm, respectively than those prepared at 1 mg mL^−1^ (274 nm) and showed a significantly (*p* < 0.01) higher antibacterial activity, as revealed by zone of inhibition diameter in Table 3.

### 2.5. Docking Studies

#### 2.5.1. Molecular Docking

Molecular docking studies were performed to identify and understand the interaction and binding affinity of Rha(s)1 and 2 with trimeric SARS-CoV-2 spike glycoproteins (S1-*N*-terminal domain (NTD) and S2 part) and the active site of different enzymes that are essential for virus replication; EndoRNAse, helicase and RNA-dependent RNA polymerase and protease. These studies were designed to address the potential application of Rha(s) for controlling and treating COVID-19 infection.

**Figure 3:**
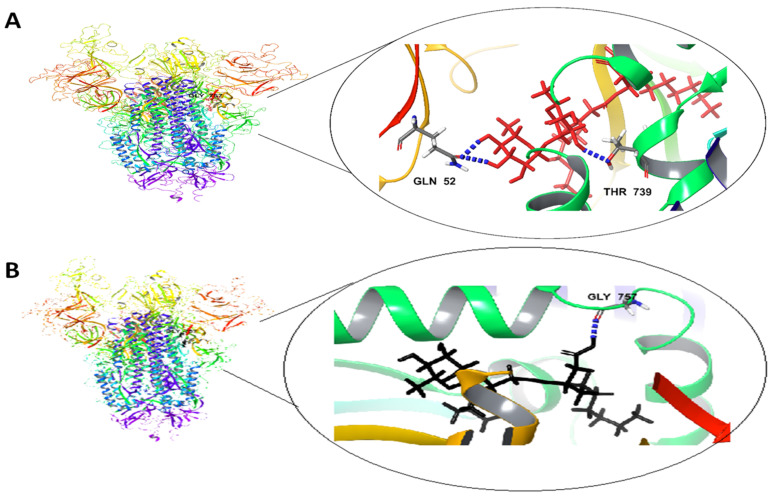
In silico docking study revealing the interactions between (**A**) Rha(s)1 and (**B**) Rha(s)2 with spike glycoproteins (S1-*N*-terminal domain (NTD) and S2 part) of SARS-CoV-2. PDB accession number for spike glycoproteins is 7CWU.

The outcome of the docking studies of Rha(s)1 and 2 with SARS-CoV-2 spike glycoproteins (S1-*N*-terminal domain (NTD) and S2 part) are presented in Figure 3 and Table 4. The binding free energy of Rha(s)1 (−45 kcal/mol) was slightly lower than Rha(s)2 (−44.6 kcal/mol) indicating the higher stability of the Rha(s)1-spike glycoproteins complex (Table 4). Thus, spike glycoproteins favored the interaction with Rha(s)1 over Rha(s)2. The interaction of Rha(s)1 with spike glycoproteins involved the formation of three hydrogen bonds with Gln 52 (chain C) and Thr 739 (chain A) and having a total intermolecular energy of −14.7 kcal/mol (Table 4). In contrast, the Rha(s)2 interaction involved the formation of just one hydrogen bond with Gly 757 (chain A) with a total intermolecular energy of −11.8 kcal/mol (Table 4).

**Figure 4:**
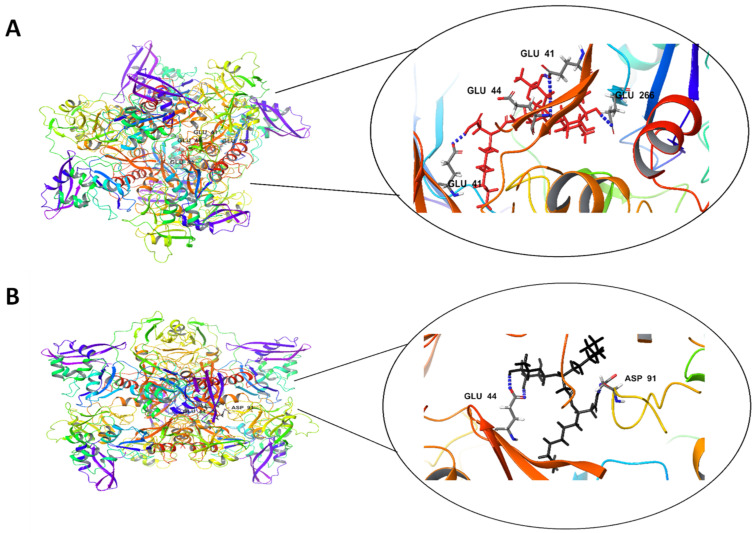
In silico docking study revealing interactions between (**A**) Rha(s)1 and (**B**) Rha(s)2 with the active sites of SARS-CoV-2 EndoRNAse. PDB accession number for EndoRNAse is 6X1B.

**Figure 5:**
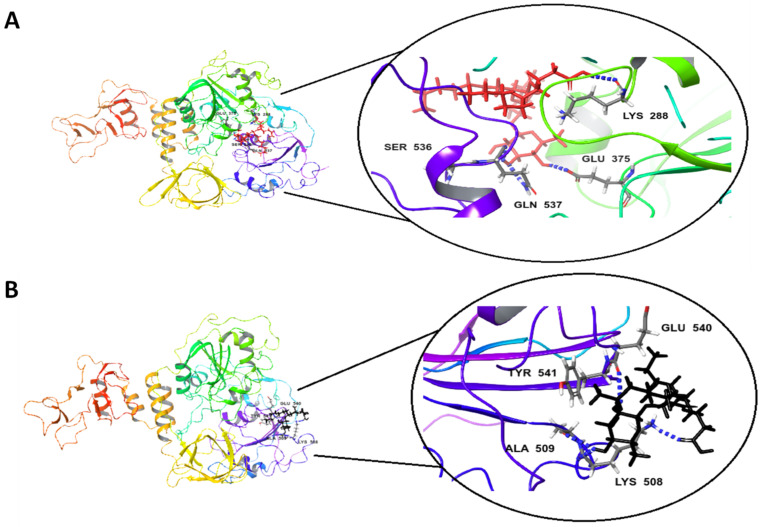
In silico docking study revealing interactions between (**A**) Rha(s)1 and (**B**) Rha(s)2 with the active sites of SARS-CoV-2 Helicase. PDB accession number for helicase is 5RL6.

**Figure 6:**
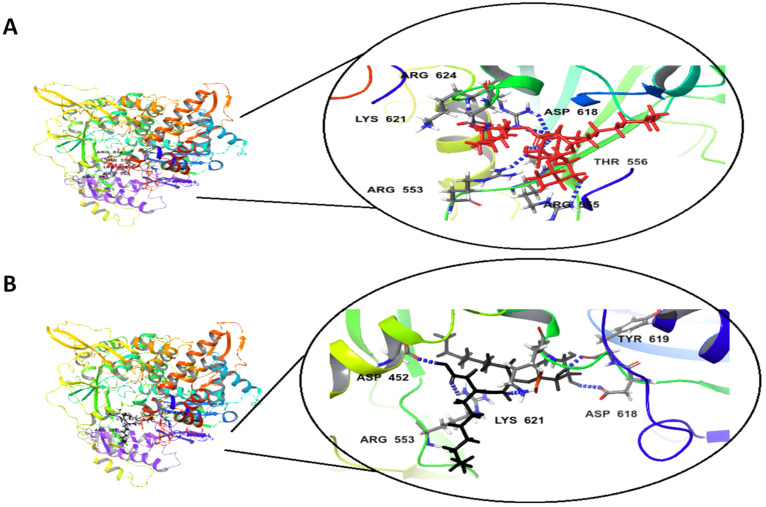
In silico docking study revealing interactions between (**A**) Rha(s)1 and (**B**) Rha(s)2 with the active sites of SARS-CoV-2 RNA-dependent RNA polymerase. PDB accession number for RNA-dependent RNA polymerase is 7CYQ.

**Figure 7:**
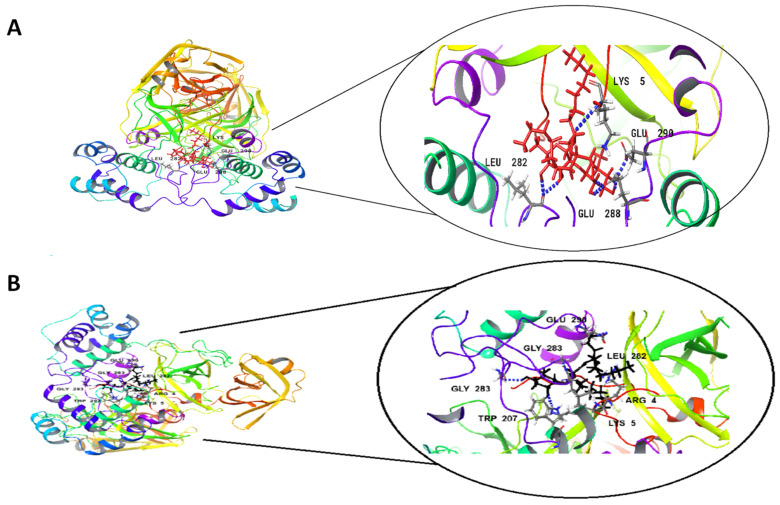
In silico docking study revealing interactions between (**A**) Rha(s)1 and (**B**) Rha(s)2 with the active sites of SARS-CoV2 main protease. PDB accession number for main protease is 6Y2G.

The docking studies of the Rha(s)1 and 2 interactions with the active sites of EndoRNAse, helicase and RNA-dependent RNA polymerase and protease are presented in Table 4 and Figure 4, Figure 5, Figure 6 and Figure 7. The binding free energy recorded with Rha(s)1 was lower than Rha(s)2 for EndoRNAse, helicase, RNA-dependent RNA polymerase and protease enzymes (Table 4). These values were −61, −66.4, −62.1 and −77 kcal/mol, respectively for Rha(s)1 versus −54, −36, −14 and −12 kcal/mol for Rha(s)2. Thus, the Rha(s)1 had a stronger interaction with the active sites of each of these enzymes than Rha(s)2.

The interactions of Rha(s)1 and 2 with EndoRNAse involved the formation of five and four hydrogen bonds, respectively. For Rha(s)1, the hydrogen bonds formed with Glu 41 chain (B), Glu 44 chain (B), Glu 41 chain (F) and Glu 266 chain (D) (Table 4 and Figure 4A), whereas for Rha(s)2, the hydrogen bonds formed with Asp 91 chain (B) and Glu44 chain (F) (Table 4 and Figure 4B). The total intermolecular free energy recorded for Rha(s)1 and 2 interactions with EndoRNAse was 21 and 11.5 kcal/mol, respectively (Table 4).

The interactions of Rha(s)1 and 2 with helicase involved the formation of three and four hydrogen bonds, respectively. For Rha(s)1, the hydrogen bonds formed with Gln 537, Glu 375, Lys 288 and Ser 536 (Table 4 and Figure 5A), whereas for Rha(s)2, the hydrogen bonds formed with Ala 509, Glu 540, and Lys 508 and Tyr 541 (Table 5 and Figure 5B). The total intermolecular free energy recorded for Rha(s)1 and 2 interactions with helicase was 14 and 5.7 kcal/mol, respectively (Table 4).

The interactions of Rha(s)1 and 2 with RNA-dependent RNA polymerase and protease involved the formation of seven and six hydrogen bonds, respectively. In the case of RNA-dependent RNA polymerase, Rha(s)1 and 2 formed hydrogen bonds with Arg 555, Arg 624, Asp 618, Thr 556 and Lys 621 and additionally with Arg 553 for Rha(s)2 (Table 4 and Figure 6A,B). For proteases, Rha(s)1 and 2 formed hydrogen bonds with Glu 288, Glu 290 and Lys 5 and additionally with Leu 282 and Gly 283 for Rha(s)1 and 2, respectively (Table 4 and Figure 7A,B).

These data imply the possibility of application of Rha(s) for treatment of COVID-19 infections and warrant investigations of Rha(s) on SARS-CoV-2 protein activity, a future perspective to be considered.

#### 2.5.2. Computational Membrane Permeability and Mode of Action of Rhamnolipids

**Figure 8:**
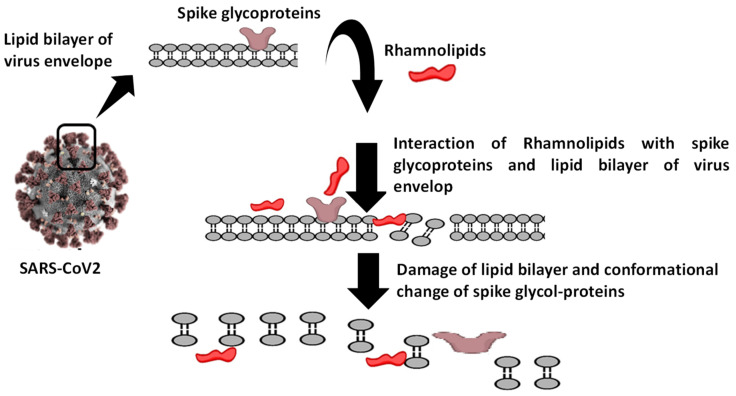
Sketch presenting the damage effect caused by rhamnolipids on the lipid bilayer of virus envelope and spike glycoproteins.

The effect of Rha(s)1 and 2 on the membrane permeability and the resultant harmful effect on the lipid envelop of SARS-CoV-2 was also investigated with the data presented in Table 5. Membrane dG Insert value of Rha(s)1 and 2 were 9.909 and 6.004, respectively, suggesting their high permeability across the viral lipid envelope (Table 5). The calculated membrane permeability values of Rha(s)1 and 2 “Log Perm RRCK” were −5.854 and −5.466 cm/s, respectively (Table 5) [34]. Log Perm RRCK of Rha(s)2 was a less negative value, which is indicative of its higher permeability compared to Rha(s)1. The resultant harmful effect of Rha(s)1 and 2 on the lipid bilayer of the virus envelope and spike glycoproteins integrity is schematically presented in Figure 8. A complete disruption of the virus envelope was expected to occur after exposure to Rha(s).

## 3. Discussion

COVID-19, a pandemic infectious disease, has caused numerous health and economic problems around the globe. Around 159 million people have been infected, and the number of deaths had exceeded 3 million across 192 countries. The high rate of viral spread is due to airborne transmission, the long asymptomatic period of the virus and its ability to remain infectious on the contaminated solid surfaces. Although, several new vaccines are currently being distributed globally, the protective effect of vaccine application globally is not yet fully clear despite its promising efficacy in clinical trials and the associated positive outcomes on disease severity. Moreover, mutated strains of SARS-CoV-2 have appeared in numerous countries, some of which can partially escape vaccine-induced immunity and will inevitably require development of updated vaccines in the future. Thus, there is still a high demand to identify a virus-specific treatment for COVID-19. Such antiviral therapies would be a promising strategy for controlling the virus spread within hospitals and among people in communities.

Proper hand hygiene was previously reported to decrease the incidence of infection in hospitals among healthcare providers. Alcohol-based sanitizers are commonly used due to their high efficacy and safety profiles, however, their misuse during the pandemic is reported to be associated with several adverse effects [7,9] as previously discussed. Therefore, finding alternative strategies for hand hygiene with less hazardous side effects are highly recommended.

The antimicrobial activity of Rha(s) was previously reported [18,26,35] and their safety profiles have been established after its topical and ocular applications [18,21]. Rha(s) are members of the glycolipid biosurfactant and they were initially found to be produced by the bacterium *Pseudomonas aeruginosa* [36]. They remain the best characterized and most frequently applied micro-organism for Rha(s) production [37,38]. Rha(s) are produced as a mixture of various ligands with highly similar structures, functions and properties [39]. As we show in Figure 1, they possess an amphiphilic property due to the composition including a hydrophilic head with one or two rhamnose sugar residues and the lipophilic lipid tail comprising one or two fatty acid residues [40,41].

In our study, a bacterial strain with a faint green fluorescent pigment, strain LeS3, was isolated from lettuce leaves. Based on rRNA sequencing data and alignment to the NCBI database, the isolate was identified as *Pseudomonas aeruginosa*. The yield of produced Rha(s) and ratios of its congeners (Rha(s)1 to Rha(s)2) depend on several factors, such as the carbon source and the fermentation conditions [38,42]. For economic purposes, there is a continuous search for finding cost effective and renewable substrates to grow *Pseudomonas aeruginosa* [43,44]. Poultry carcass yields are typically about 70–75% of the live bird weight and is one of the waste products resulting from the poultry industry [45]. Carcasses contain a high content of organic matter [46] and this renders them good substrates for use in the production of important products instead of their disposal by conventional methods. In our study, Rha(s) were produced by growing *Pseudomonas aeruginosa* strain LeS3 in a new medium formulated from chicken carcass soup (CCS) in a trial designed to optimize the yield and the ratio of Rha(s)1 to 2. To the best of our knowledge, this is the first study using broth media wholly formulated from CCS for this purpose.

Our results indicated that Rha(s) yield was increased by 5 times with CCS medium compared to the commonly used medium, (GSNB), which may be due to the presence of growth factors supporting the growth of the bacterium and subsequent production of Rha(s). We are assuming that the presence of some amino acids, vitamins and different minerals might be responsible for such increment and this was matched with what was reported previously when some byproducts such as molasses and whey were used for the production of Rha(s) using *Pseudomonas aeruginosa* [43,47]. However further studies are currently being investigated to figure out the role of different factors using such medium in increasing Rha(s) yield.

The presence of a hydrophobic carbon source (chicken fats) favored the dominance of Rha(s)1 over Rha(s)2 as revealed by ESI-MS analysis shown in Section 2.2.1. Our results match those previously reported [42,48,49] where Rha(s)1 were the dominant compounds produced when the bacteria were grown in a medium supplemented with hydrophobic carbon sources, whereas using hydrophilic carbon sources favored the dominance of Rha(s)2.

In our study, R-C_8:2_ with the polyunsaturated β-hydroxy-fatty acid chains was the predominant mono-rhamnolipid congener identified and this has been identified previously [50]. In contrast to that reported by Nitschke and his colleagues [51], where Rha(s)1 (R-C_10_C_10_) was the main component of Rha(s) produced when a hydrophobic carbon source was used for growing *Pseudomonas* strains, while hydrophilic carbon sources lead to predominance of the Rha(s)2 such as (R-R-C_10_C_10_).Therefore, the new cost effective medium (CCS) used in our study was enriched with a hydrophobic carbon source and favored the production of Rha(s)1. As was previously presented, Rha(s)1 showed a better antiviral activity than Rha(s)2 against SARS-CoV-2.

Nanotechnology application is a growing field where it is interested in production of fibers [52] and particles in nanometer scale to improve the therapeutic activity, reduce side effects of medicines and could be also used for diagnostic purposes [53,54]. Currently nanotechnology is applied to combat the current COVID-19 pandemic [55,56,57]. Nanoparticles (NPs) have been applied previously for the treatment of several viral infections with promising results [27,54,58,59,60] and they were also demonstrated a good antibacterial activity against multidrug resistant bacteria [61,62,63]. Therefore, the previously isolated and characterized Rha(s) mixture was used to produce Rha(s) nano-micelles containing different concentrations of rhamnolipids (1, 5 and 10 mg mL^−1^). The formed nano-micelles ranged in size from 164 to 274 nm as measured by Malvern Zeta sizer. TEM images obtained for samples prepared at 5 mg mL^−1^ showed spherical nano-micelles with no signs of aggregation but with a reduced size than that recorded by Malvern zeta sizer. This difference most likely reflects variations of size discrimination by these techniques. All samples produced were stable as revealed from Zeta potential values (≥−50). Importantly, samples were monodispersed with a lower tendency to aggregate as indicated by PDI value (PDI ≤ 0.3). However, those samples prepared at 1 mg mL^−1^ showed greater aggregation properties. This is consistent with other studies reporting that PDI < 0.3 [64,65,66] is indicative of good homogeneity and to be suitable for drug delivery applications. Whereas another study revealed that nano-micelles of PDI > 0.3 is indicative of a highly polydisperse sample [67].

The concentration and size dependent antibacterial activity of Rha(s) nano-micelles against selected Gram-negative and Gram-positive drug resistant bacterial strains was demonstrated by the agar well diffusion method. By increasing the concentration and decreasing the size of nano-micelles, an increase of antibacterial activity against both Gram-positive and Gram-negative bacteria was noted with MIC values of 0.031 mg mL^−1^ and >0.5 mg mL^−1^ for Gram-positive and Gram-negative tested strains, respectively. The improvement of antibacterial activity by decreasing the size of nano-micelles might be attributed to the larger effective surface area offered by smaller nano-micelles for interaction with the bacteria.

The antibacterial activity of Rha(s) could be due to their solubilizing effect on the phospholipid bilayer of the bacteria, thereby increasing the permeability and flow out of metabolites. Such a change in phospholipid bilayer structure and function was previously reported to affect protein conformation, transport and energy generation, ultimately leading to bacterial cell death [49].

The antimicrobial activity of Rha(s) is affected by the Rha(s) 1 to Rha(s) 2 ratio [23]. Our results revealed a higher antibacterial activity against Gram-positive bacteria that contrasts with what was reported by Das and his colleagues [68] who found that changing the proportion of Rha(s)1 to Rha(s)2 is accompanied with a change of the antimicrobial activity of Rha(s) mixture where an increase of mono to di species, the Rha(s) mixture becomes more effective against Gram-negative bacterial strains. It was concluded that an increase of emulsification index could enhance the penetration of Rha(s) across the cell wall of Gram-negative bacteria, resulting in death. Our data supports what was commonly reported in the literature where Rha(s) showed greater antibacterial activity against Gram-positive over Gram-negative bacteria [23,69,70,71]. The reduced effect on Gram negative bacteria could be attributed to the presence of the lipopolysaccharide outer membrane that confers protection to the cell. Similarly, de Freitas Ferreira and colleagues [70] reported a predominance of Rha(s)1 to Rha(s)2 by a ratio of 2:1, also revealed a resistance of Gram-negative strains. This is contrary to the Rha(s) mixture produced by *P. aeruginosa* 47T2 with the predominance of Rha(s)2 homologs and was reported to inhibit the growth of *Escherichia coli* and *Salmonella* Typhimurium with MIC values of 64 μg mL^−1^ and 128 μg mL^−1^ respectively [18]. Therefore, the differences observed in the sensitivity of Gram-negative strains to Rha(s) might be dependent not only on the composition of biosurfactant and its purity but also on the bacterial adaptation ability [72]. The nutritional and environmental conditions might also have additional influence on the antimicrobial activity as previously reported [70].

A molecular docking study was also performed to investigate the potential antiviral activity of Rha(s) against SARS-CoV-2. Compounds that interact with the SARS-CoV-2 spike glycoproteins (S1-*N*-terminal domain (NTD) and S2 part) are hypothesized to interfere with virus attachment to host entry receptors (ACE-2) with a consequent loss of viral infectivity. As revealed from our docking studies, Rha(s)1 and 2 interact efficiently with the SARS-CoV-2 spike glycoproteins with Rha(s)1 forming the most stable complex as indicated by its lower free energy. These interactions are expected to result in irreversible changes to the virus spike proteins structure and inhibition of viral infectivity. Our data also showed the ability of Rha(s) as a biosurfactant to interact with the lipid membranes (lipid envelope) of SARS-CoV-2, which is expected to be associated with disruption of membrane permeability similar to that previously observed for HSV1 and HSV2 [73,74,75,76]. Taken together, Rha(s) could therefore be a promising agent to control the spread of COVID-19 infection and warrants further in vitro study of viral infection. Ultimately, Rha(s) could be used by patients with COVID-19 and health care providers as a hand sanitizer in the current pandemic.

Further docking studies were performed to understand the interaction of Rha(s)1 and 2 with the active sites of enzymes involved in virus replication. These included four key viral proteins including EndoRNAse, helicase, RNA-dependent RNA polymerase and the main protease. Our results showed that the free energy from interaction of Rha(s)1 with these enzymes was lower than that obtained with Rha(s)2. These Rha(s) interactions were expected to cause detrimental conformational changes at the active binding sites of these enzymes rendering them inactive in virus replication. Therefore, Rha(s) nano-micelles might be recommended for treatment of COVID-19 infection, however, their safety profile must be addressed and further in vitro studies confirmed.

**Figure 9:**
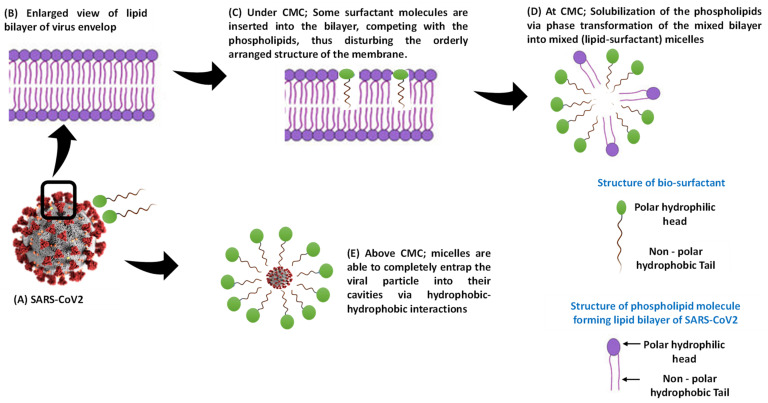
Sketch presenting the inactivation of SARS-CoV-2 by biosurfactants as a single molecules and nano-micelles.

Although the docking studies revealed the interactions between Rha(s) as a single molecule with SARS-CoV-2 proteins, these interactions could however also be applied to Rha(s) nano-micelles. The proposed interactions of surfactant as a single molecule or in the form of nano-micelles has been previously reported [77,78] and is presented here in Figure 9. The interactions of surfactant either side of the critical micelle concentration (CMC) with SARS-CoV-2 were previously reported [77]. Below CMC, it was reported [79] that the phospholipid in the bilayer and the surfactant monomers interact via hydrophobic interactions between the lipid tails and the surfactant tails. Molecules of the added surfactants are also inserted into the bilayer, competing with the phospholipids, thus disturbing the orderly arranged structure of the membrane. When the surfactant concentration approaches the CMC, the lipid–surfactant mixed bilayers become saturated and no longer accommodate additional surfactants. This induces solubilization of the phospholipids via phase transformation of the mixed bilayer into mixed (lipid–surfactant) micelles [80]. Above CMC, when the surfactant-to-lipid concentration ratio increases, micellization is completed, i.e., the lipid bilayer is completely solubilized by the surfactants and only the micellar aggregates remain in the solution [81]. Thus, the complete solubilization of the protective lipid bilayer leads to the potential disintegration of the virus into fragments, neutralizing infectivity. Alternatively, micelles are able to completely entrap the viral particle internally via hydrophobic–hydrophobic interactions [77]. Based on these finding, Rha(s) nano-micelles show a high potential for use against SARS-CoV-2 as a hand sanitizer to combat the current pandemic infections.

## 4. Materials and Methods

### 4.1. Materials

Microbiological media (tryptone soy broth; TSB, tryptone soy agar; TSA, Muller Hinton agar; MHA, and Muller Hinton broth; MHB) were purchased from HiMedia, Mumbai, India. All chemicals and reagents were of analytical grade. Peptone and sodium chloride were purchased from Oxoid, Cheshire, UK. Hydrochloric acid, ethyl acetate and sulphuric acid were purchased from Honeywell™, North Carolina, USA. L- rhamnose was purchased from Sigma-Aldrich, Cairo, Egypt. Orcinol was obtained from SDFCL, Kolkata, India.

### 4.2. Methods

#### 4.2.1. Isolation of Biosurfactant Producing Bacterial Strain

The bacterial strain used in this study was isolated from lettuce leaves collected from an open field in Giza Governorate, Egypt. Briefly, 25 g of collected lettuce leaves were washed with sterile water to remove soil particles then the leaves were homogenized with a solution composed of 0.1% peptone and 0.85% sodium chloride. A total of 100 µL of the homogenized mixture was streaked on tryptic soy plates and incubated overnight at 37 °C. Colonies with a halo of faint green fluorescent pigment were picked up, marked as LeS3 and maintained on TSA slants.

#### 4.2.2. Identification of Biosurfactant Producing Bacterial Strain

Identification of the bacterial isolate was based on morphological and molecular characteristics. Polymerase chain reaction (PCR) was performed using 27F, 1492R 16S rRNA primers and Mytaq Red DNA polymerase master mix (BIOLINE cat # BIO-21108) according to the manufacturer’s instructions. The amplification reactions were performed in a Thermal Cycler (Biometra, Germany) at 50 °C annealing temperature following a previously published protocol [82]. The PCR product was purified using PCR-M clean up system (VIOGENE cat# PF1001) according to the manufacturer’s protocol. The purified cDNA sequence was obtained by GATC Company using ABI 3730xl DNA sequencer using the 27F-16S rRNA primer. The nucleotide sequences were aligned to the total bacterial nucleotide collection of NCBI using the basic local alignment search tool of nucleotide blast (https://blast.ncbi.nlm.nih.gov/Blast.cgi), accessed date 18 January 2020 and released date 23 January 2020).

#### 4.2.3. Production and Characterization of Rha(s)

##### Production of Rha(s)

Rha(s) production was carried out following a literature protocol using shake flask technique and separated from the production medium by acid precipitation followed by organic solvent extraction [83]. New production medium formulated from chicken carcass soup (CCS) containing 5% chicken fat, 0.5% NaCl was compared with glycerol supplemented nutrient broth (GSNB) (nutrient broth medium containing 10 g L^−1^ glycerol) to optimize the yield of Rha(s). Briefly, *Pseudomonas aeruginosa* strain LeS3 was grown in TSB to obtain OD_600_ of 0.8, which corresponds to a density of 8 log cfu mL^-1^ A 250 mL Erlenmeyer flask containing 100 mL of either sterilized GSNB or CCS was then inoculated with 1% of prepared bacterial culture. Inoculated flasks were incubated in the orbital shaker (Vision Scientific Co., Ltd. Korea. VS-8480SR) at 30 °C and 150 rpm for 5 days.

Following the incubation period, bacterial cells were removed from the culture broth by centrifugation at 10,000 rpm at 5 °C for 10 min (Sigma, Germany, 3-16PK) to obtain cell free supernatant (CFS). The CFS was acidified to pH 2.0 using 1N HCl and stored overnight at 5 °C. Rha(s) were then extracted using an equal volume of ethyl acetate. The yellow/brown viscous paste was then stored at 4 °C until further characterization.

Quantification of Rha(s) was carried out using the orcinol assay [83], 333 µL of CFS was extracted twice with ethyl acetate and evaporated followed by the addition of 0.5 mL distilled water. The assay mixture consisted of freshly prepared reagent containing 0.19% orcinol in 53% sulphuric acid that was added to the extracted biosurfactant in the ratio of 9:1. This mixture was heated in a water bath at 80 °C for 30 min and allowed to cool to room temperature before measuring its optical density at 421 nm. The Rha(s) concentrations were calculated from a standard curve prepared with L-rhamnose and expressed as rhamnose equivalents (RE) by multiplying rhamnose values by a coefficient of 3.4 obtained from the correlation of pure Rha(s)/rhamnose [84].

##### Characterization of Rha(s)

ESI-MS Analysis of Rha(s) Mixture

A mixture of Rha(s) (crude extract) was prepared at a concentration of 100 μg mL^−1^ for ESI-MS analysis. Chromatographic separation was performed on an Acquity UPLC system (BEH C18 Column, 130 Å, 1.7 µm, 2.1 mm × 50 mm) with gradients elution of MeOH/H2O at a flow rate of 0.2 mL min^−1^. The column temperature was maintained at 25 °C and the injection volume was 10 μL. The UPLC is coupled to an online PDA and MS detector. The ESI-MS analysis in both positive and negative ion mode was carried out on a XEVO TQD triple quadruple instrument (Waters Corporation, Milford, MA 01757, USA, mass spectrometer).

Data Processing

The peaks and spectra were processed using the Maslynx 4.1 software and tentatively identified by comparing its retention time (Rt) and mass spectrum with reported data.

#### 4.2.4. Preparation and Characterization of Rha(s) Nano-Micelles

Rha(s) nano-micelles were prepared using different concentrations (1, 5 and 10 mg mL^−1^) of Rha(s) crude extract that were dispersed in PBS, pH7.4 using a probe sonicator for 3 min. Particle size and zeta potential were determined using a Malvern Zeta-sizer Nano ZS (Malvern Instruments Ltd., Malvern, UK) at 25 ± 0.1 °C.

Rha(s) nano-micelles were imaged by TEM (H-700, Hitachi Ltd., Japan), at an accelerated voltage of 80 kv using the negative staining method. The solution of Rha(s) nano-micelles was diluted (1:50) with distilled water then a drop of the diluted solution was spread on a mesh copper grid coated with carbon film and was kept for 5 min to dry. Then after, a drop of phospho-tungstic acid (2% *w*/*w*) was added on the grid for 50 s, before excess liquid was removed using filter paper.

#### 4.2.5. The Antibacterial Activity of Rha(s)

The antibacterial activity of produced Rha(s) was evaluated against selected multi drug resistant strains, *Streptococcus pneumoniae*, *Staphylococcus aureus*, *Salmonella* Montevideo and *Salmonella* Typhimurium using agar well diffusion method according to a published protocol [85]. Briefly, Mueller–Hinton agar plate surface was spread with a volume of freshly prepared microbial inoculum containing 5 log cfu mL^−1^. Then, a hole with a diameter equivalent to 7 mm was punched under aseptic conditions using a sterile blue tip base. Then, the formed wells were filled in with 100 µL of the previously prepared Rha(s) nano-micelles colloidal solutions. Following inoculation, plates were incubated at 37 °C for 24 h and examined for bacterial growth and clear zone formation. Minimum inhibitory concentration (MIC) of Rha(s) against specific bacterial strains was determined using a microdilution assay [86]. Briefly, two-fold serial dilutions of Rha(s) (ranging from 1 to 0.0195 mg mL^−1^) in MHB were prepared and added to 96 well plates. Wells were then inoculated with 10 µL of 5 log cfu mL^−1^ (final inoculum) of bacteria. Inoculated plates were then incubated for 18–24 h at 37 °C. The MIC value was defined as the lowest concentration that inhibited visible growth.

#### 4.2.6. Docking Study

For the COVID-19 in silico study, the chemical and 3D structure of Rha(s)1 and Rha(s) 2 presented in Figure 1 were obtained from the PubChem database (https://pubchem.ncbi.nlm.nih.gov, accessed on 16 June 2021). The Protein Data Bank (PDB) database (https://www.rcsb.org/, accessed on 16 June 2021) was used to obtain the complete 3D structure of SARS-CoV-2 EndoRNAse (PDB accession number: 6X1B), helicase (PDB accession number: 5RL6), RNA-dependent RNA polymerase (PDB accession number: 7CYQ), spike glycoproteins (PDB accession number: 7CWU) and protease enzyme (PDB accession number: 6Y2G)

##### In Silico Molecular Modelling

The interaction of rhamnolipids, Rha(s)1 and 2 with SARS-CoV-2 spike glycoproteins and enzymes involved in viral replication: EndoRNAse, helicase, RNA-dependent RNA polymerase and protease were studied using in silico docking. The ligands binding studies were carried out using Maestro 11.9 software. Energy minimization of ligand was optimized and ligand preparation was performed using LigPrep 2.4 software. Docking of ligands was achieved using Schrodinger Maestro 11.9 software and Glide’s Extra Precision (XP) [87]. The size of grid box for each protein was set to 20 Å by default.

#### 4.2.7. Statistical Analysis

Statistical analysis was done by Minitab version 17 at a confidence level of 95% using a two-way ANOVA.

## 5. Conclusions

Rha(s) isolated from a novel strain of *Pseudomonas aeruginosa* strain LeS3 were shown to form a variety of typical structures with the congener R-C_8:2_ containing the polyunsaturated β-hydroxy-fatty acid chains predominating. Rha(s) nano-micelles prepared from a Rha(s) mixture had a size ranged from 164 ± 1 to 274 ± 50 nm and by increasing Rha(s) concentration ≥5 mg mL^−1^, a monodisperse sample was obtained as revealed by a PDI value that was ≤0.3. Rha(s) nano-micelles showed antibacterial activity against both Gram-positive (*Streptococcus pneumoniae* and *Staphylococcus aureus*) and Gram-negative *(Salmonella* Montevideo and *Salmonella* Typhimurium) human bacterial pathogens. The antibacterial activity was recorded to be size and concentration dependent with preferred antibacterial activity against Gram-positive bacteria where MIC (0.031 mg mL^−1^) with Gram-positive bacteria was significantly (*p* < 0.05) lower than its value (>0.5 mg mL^−1^) for Gram-negative bacteria. The molecular docking studies showed interactions with SARS-CoV-2 structural and non-structural proteins. Computational studies also demonstrated membrane permeability activities suggesting that Rha(s) nano-micelles have multiple mechanisms of action and could be applied as antiviral agents in addition to their antibacterial function. Therefore, Rha(s) nano-micelles could be recommended to replace alcohol-based hand sanitizer in general communities and health care settings under the current pandemic infection of COVID-19. Furthermore, Rha(s) nano-micelles might be also recommended to be investigated in the treatment of COVID-19. However, further studies should be performed to address their antiviral effects and safety profiles. These additional studies are currently underway to establish its antiviral activity and development as interventions for COVID-19.

## Figures and Tables

**Table 1:** Congeners composition of rhamnolipids mixture produced by *P. aeruginosa* strain LeS3 as analyzed by LC/ESI-MS at both positive and negative modes.

Rha(s) Congeners	*m/z*							
	Mol f	Mol wt	[M-H]^−^	[M+H]^+^	[M+Na]^+^	[M+K]^+^	[M-H+Na_2_]^+^	% Abundance
**Mono-Rhamnolipid (Rha(s)1) Congeners**								
**R-C_8_**	C_14_H_26_O_7_	306					351	12.8
**R-C_8:1_**	C_14_H_24_O_7_	304			327			25.2
**R-C_8:2_**	C_14_H_22_O_7_	302			325			29.4
**R-C_9:1_**	C_15_H_26_O_7_	318			341			0.3
**R-C_10_**	C_16_H_30_O_7_	334			357		379	13.6
**R-C_10:2_**	C_16_H_26_O_7_	330			353			0.12
**R-C_12_**	C_18_H_34_O_7_	362			385			0.24
**R-C_12:2_**	C_18_H_30_O_7_	358		359	381			2.28
**R-C_13_**	C_19_H_36_O_7_	376					421	0.56
**R-C_13:2_**	C_19_H_32_O_7_	372			395			0.8
**R-C_14_**	C_20_H_38_O_7_	390			413			0.06
**R-C_15_**	C_21_H_40_O_7_	404				443		0.24
**R-C_8_ -C_12_, R-C_9_-C_11_, R-C_10_-C_10_, R-C_12_ -C_8_, R-C_11_-C_9_**	C_26_H_48_O_9_	504	503		527	543		6.8
**R-C_8_-C_14_, R-C_9_-C_13_, R-C_10_-C_12_, R-C_11_-C_11_**	C_28_H_52_O_9_	532	531				577	0.52
**R-C_8_-C_14:1_, R-C_9_-C_13:1_, R-C_10_-C_12:1_, R-C_11_-C_11:1_** **, R-C_8:1_-C_14_, R-C_9:1_-C_13_, R-C_10:1_-C_12_, R-C_11:1_-C_11_**	C_28_H_50_O_9_	530			553			0.6
**R-C_11_-C_16_, R-C_12_-C_15_, R-C_13_-C_14_**	C_33_H_62_O_9_	602		603		641		0.52
**R-C_14_-C_16:2_, R-C_15_-C_15:2_, R-C_14:2_-C_16_, R-C_15:2_-C_15_**	C_36_H_64_O_9_	640		641				0.8
**Di-rhamnolipid (Rha(s)2) congeners**								
**R-R-C_12:1_**	C_24_H_42_O_11_	506					551	0.22
**R-R-C_16:1_**	C_28_H_50_O_11_	562				601		0.62
**R-R-C_8_-C_10:2_, R-R-C_9_-C_9:2,_ R-R-C_8:2_-C_10_, R-R-C_9:2_-C_9_**	C_30_H_50_O_13_	618			641	657		1.42
**R-R-C_8_ -C_12_, R-R-C_9_ -C_11_, R-R-C_10_ -C_10_, R-R-C_12_-C_8_, R-R-C_11_ -C_9_,**	C_32_H_58_O_13_	650	649		673			1.6
**R-R-C_16_-C_16:2_** **, R-R-C_16:2_-C_16_**	C_44_H_78_O_13_	815		816				0.03
**Mol F, Molecular formula** **Mol wt, Molecular weight** **R, Rhamnose**								

**Table 2:** Characterization of Rha(s) nano-micelles prepared at different concentrations; 1, 5 and 10 mg mL^−1^ in PBS buffer.

Concentrations of Rhamnolipids (mg mL^−1^)	Particle Size (D nm ± SD)	Polydispersity Index (PDI)	Zeta Potential (mv ± SD)
1	274 ± 50	0.55	−50.4 ± 1.7
5	164 ± 1	0.30	−62.07 ± 3.8
10	169 ± 10	0.27	−66.77 ± 2.62

**Table 3:** The antimicrobial activity of rhamnolipids nano-micelles was presented as average ± standard deviation. Results are average of two independent experiments with three replicates in each.

Bacterial Strain	Concentration of Rha(s) (mg mL^−1^)			
	MIC	1	5	10
		Corresponding Zone of Inhibition (mm) ± SD		
**Gram-positive bacteria**				
*Streptococcus pneumoniae*	0.031	9.6 ± 1.2	16.5 ± 1	23 ± 2
*Staphylococcus aureus*	0.031	17.8 ± 0.76	25 ± 1	30 ± 1.5
**Gram-negative bacteria**				
*Salmonella* Montevideo	>0.5	7.1 ± 1	15 ± 1	21 ± 1.5
*Salmonella* Typhimurium	>0.5	6.8 ± 0.76	12.1 ± 1.2	18.1 ± 1.7

**Table 4:** The docking interaction parameters of both Rha(s)1 and Rha(s)2 with spike glycoproteins of SARS-CoV-2 and enzymes involved in viral replication: EndoRNAse, helicase, RNA-dependent RNA polymerase and protease.

Ligands	Binding Free Energy (kcal/mol)	Total Intermolecular Energy (kcal/mol)	Interacting Amino Acids	Hydrogen Bonds
**Spike glycoproteins**				
**Rha(s)1**	−45	14.7	Gln 52 and Thr 739	3H bonds
**Rha(s)2**	−44.6	11.8	Gly 757	1H bonds
**EndoRNAse**				
**Rha(s)1**	−61	20.5	Glu 41, Glu 44 and Glu 266	5H bonds
**Rha(s)2**	−53.9	11.5	Asp, Glu44, and Lys 46	4H bonds
**Helicase**				
**Rha(s)1**	−66.4	14.2	Gln 537, Glu 375, and Lys 288	3H bonds
**Rha(s)2**	−35.5	5.7	Asp542, Glu 540, and Lys 508	4H bonds
**RNA-dependent RNA polymerase**				
**Rha(s)1**	−62.1	17.3	Arg 555, Arg 624, Asp 618, Thr 556 and Lys 621	7H bonds
**Rha(s)2**	−55.8	13.7	Arg 555, Arg 624, Asp 618, Thr 556, Arg 553, and Lys 621	6H bonds
**Protease**				
**Rha(s)1**	−77	22.1	Glu 288, Glu 290, Leu 282 and Lys 5	7H bonds
**Rha(s)2**	−61.1	12.1	Glu 288, Glu 290, Gly 283 and Lys 5	6H bonds

**Table 5:** Computational membrane permeability of rhamnolipids (Rha(s)).

Ligand	Membrane Permeability Prediction					
	^1^ Membrane *dG Insert	^2^ Membrane HDLD	^3^ Membrane GB	^4^ Membrane State Penalty	^5^ Log Perm RRCK (cm/s)	Membrane Energy
**Rha(s)1**	9.909	5.516	−3.033	9.909	−5.854	13.416
**Rha(s)2**	6.004	1.610	−6.789	6.004	−5.466	−1.146

* Partition energy “dG” Insert prediction; ^1^ Membrane dG Insert: the total free energy penalty for the ligand to change state and enter the membrane. This is the net of the energy of Membrane HDLD and Membrane State Penalty; ^2^ Membrane HDLD: the free energy penalty for the neutral form of the ligand in its conformation inside the membrane to enter the membrane (i.e., move from the high dielectric region to the low dielectric region, hence HDLD). ^3^ Membrane GB: an implicit membrane generalized born theory model closely reproduces the Poisson–Boltzmann (PB) electrostatic solvation energy profile across the membrane. ^4^ Membrane State Penalty: a tautomerization penalty is derived from possible tautomer states and their estimated relative populations. These two processes are combined as a state penalty, ΔG state, that represents the free energy cost for the permeant to adopt a particular neutral, tautomeric form for membrane permeation. ^5^ Log Perm RRCK: logarithm of the RRCK permeability in cm/s. This property is optimized to reproduce RRCK permeability assay results, with fitted energy.

## Data Availability

All authors are happy to share all data (including supplementary data) presented in this manuscript to the public repository.

## Supplementary Materials

The following are available online at https://www.mdpi.com/article/10.3390/antibiotics10070751/s1, Figure S1: PCR amplification products of 16S-rRNA encoding genes of *Pseudomonas aeruginosa* strain LeS3 showed a fragment length of 1500 bp. (1 kb+) refers to DNA ladder.
