# Supplementary material for: Rhamnolipids Nano-Micelles as a Potential Hand Sanitizer
Source: Antibiotics (Basel). 2021 Jun 22;10(7):751. doi: 10.3390/antibiotics10070751 (PMC8300634; doi:10.3390/antibiotics10070751)
Supplement: Supplementary file 1 [file antibiotics-10-00751-s001.zip › antibiotics-1117872-supplementary.pdf]

antibiotics

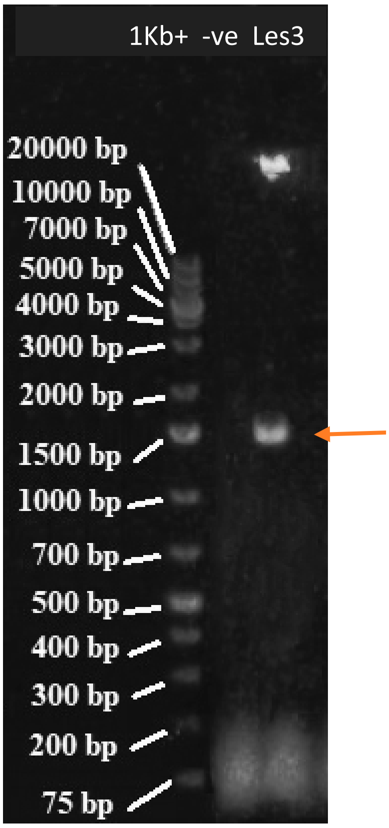

Figure S1. PCR amplification products of 16S-rRNA encoding genes of *Pseudomonas aeruginosa* strain LeS3 showed a fragment length of 1500 bp. (1Kb +) refers to DNA ladder. (-ve) refers to negative control: no amplification confirms no PCR contamination.
